# Differential age-dependent development of inter-area brain connectivity in term and preterm neonates

**DOI:** 10.1038/s41390-022-01939-7

**Published:** 2022-01-29

**Authors:** Takeshi Arimitsu, Naomi Shinohara, Yasuyo Minagawa, Eiichi Hoshino, Masahiro Hata, Takao Takahashi

**Affiliations:** 1 Department of Pediatrics, Keio University School of Medicine, Shinjuku, Tokyo, 160-8582 Japan; 2 Department of Psychology, Faculty of Letters, Keio University, Kohoku-ku, Yokohama, 223-8521 Japan; 3 Keio University Global Research Institute, Minato-ku, Tokyo, 108-8345 Japan

## Abstract

**Background:**

Among preterm infants, higher morbidities of neurological disturbances and developmental delays are critical issues. Resting-state networks (RSNs) in the brain are suitable measures for assessing higher-level neurocognition. Since investigating task-related brain activity is difficult in neonates, assessment of RSNs provides invaluable insight into their neurocognitive development.

**Methods:**

The participants, 32 term and 71 preterm neonates, were divided into three groups based on gestational age (GA) at birth. Cerebral hemodynamic activity of RSNs was measured using functional near-infrared spectroscopy in the temporal, frontal, and parietal regions.

**Results:**

High-GA preterm infants (GA ≥ 30 weeks) had a significantly stronger RSN than low-GA preterm infants and term infants. Regression analyses of RSNs as a function of postnatal age (PNA) revealed a steeper regression line in the high-GA preterm and term infants than in the low-GA infants, particularly for inter-area brain connectivity between the frontal and left temporal areas.

**Conclusions:**

Slower PNA-dependent development of the frontal–temporal network found only in the low-GA group suggests that significant brain growth optimal in the intrauterine environment takes place before 30 weeks of gestation. The present study suggests a likely reason for the high incidence of neurodevelopmental impairment in early preterm infants.

**Impact:**

- Resting-state fNIRS measurements in three neonate groups differing in gestational age (GA) showed stronger networks in the high-GA preterm infants than in the term and low-GA infants, which was partly explained by postnatal age (PNA).
- Regression analyses revealed a similar PNA-dependence in the development of the inter-area networks in the frontal and temporal lobes in the high-GA and term infants, and significantly slower development in the low-GA infants.
- These results suggest that optimal intrauterine brain growth takes place before 30 weeks of gestation. This explains one of the reasons for the high incidence of neurodevelopmental impairment in early preterm infants.

## Introduction

Advancements in perinatal medicine have improved the survival rate of preterm infants, which has resulted in an increased population of preterm infants worldwide. This trend has continued in a large number of preterm births.^1^ Higher morbidity of neurological disturbance and developmental delay of either perceptual or cognitive function have become a critical issue among preterm infants.^2–4^ To solve this problem, early detection and intervention are essential.^5,6^ Previous research has demonstrated that various states of nervous system disorders (e.g., intraventricular hemorrhage (IVH), periventricular leukomalacia (PVL), and severe bronchopulmonary dysplasia) or some indices such as head circumference predict the neurological prognosis of preterm infants. However, there is no definite index that directly reflects this, particularly for the development of higher brain functions. In this situation, the direct measurement of brain function and establishment of milestones in early infancy is necessary. One possible milestone of brain development is the development of a resting-state network (RSN) in preterm infants, because it reflects the brain network of cognitive function as stated below and could also be utilized for the assessment of neonates.^7–10^

Accumulating evidence has demonstrated that not only focal cerebral activity but also connections between various cerebral areas are important for the implementation of higher brain functions.^11,12^ The connectivity of the cerebral network in preterm infants is different from that in term infants, and such a difference has been reported to correlate with neurological disturbances in preterm infants.^7,13–16^ RSN is an intrinsic cerebral connectivity that does not process stimulations or cognitive tasks. In the absence of exogenous stimulation, cerebral neuronal activity spontaneously consumes 20% of the body’s energy, despite representing only 2% of the total body mass. Spontaneous neuronal activity mainly maintains ongoing neuronal signaling, which forms a large-scale, coordinated connectivity network.^17,18^ Intrinsic cerebral connectivity is mostly based on structurally connected regions that engage in spontaneous neuronal activity via white matter axons.^12,19–23^ However, the RSN is not always constrained by anatomical structures,^24^ rather it reflects a functional network that is responsible for various types of cognitive functions. Specifically, those functions include preparing for task execution (executive control network), continuous monitoring of external and internal environments (default-mode network), mind wandering, and memory consolidation.^19,25–27^ The RSN is considered biologically important for higher-level cognition^17,28^; therefore, RSN has been proposed as a useful biomarker of various cognitive impairments such as attention deficit.^9,10,29^ Since it is difficult to investigate task-related studies in neonates in the same way as it is done in adults, studies assessing RSNs in neonates provide invaluable insights.

A decade ago, the number of studies using magnetic resonance imaging (MRI) to investigate RSNs in preterm infants has increased. The development of RSNs in preterm infants is different from that in term infants. The RSN of preterm infants is underdeveloped, and it develops differently depending on the cerebral region and postmenstrual age (PMA) of infants.^18,30–35^ Preterm infants at the expected date of birth presented weak or unorganized RSN compared to term infants at term-equivalent age.^27,32,35–40^ Kwon et al.^30^ demonstrated decreased lateralization in the left frontotemporal language areas, decreased interhemispheric and intrahemispheric connections, and increased connectivity between the left Brodmann area (BA) 22 and right BA 39 in preterm infants than in term infants. Smyser et al.^36^ demonstrated a PMA-dependent increase in interhemispheric connections between homotopic counterparts in the sensorimotor cortex.^32,36^ Another study also demonstrated a PMA-dependent increase in local clustering and shortest path length.^33^ These developments differ among brain regions. Cao et al.^33^ revealed differential PMA-dependent development among brain regions by demonstrating increased connectivity in the primary motor, somatosensory, visual, and auditory regions in contrast to a decreased connectivity in high-order default mode and executive control regions. Studies on fetuses have demonstrated similar results to those of preterm infants. In fetal functional MRI (fMRI) studies, long- and short-range RSN increased with PMA, and connectivity developed from medial to lateral and from posterior to anterior.^31,41–43^ Thomason et al.^44^ demonstrated reduced connectivity of the left hemispheric language region, and this interhemispheric connectivity has been demonstrated prenatally in preterm-born infants. The clinical course also affects the development of RSN in the brain.^45^ Thus, assessing RSNs in preterm infants can reveal early neurological development, including regions responsible for language, motor, and executive functions, which could facilitate early detection and intervention for developmental delay.^8,46^

As summarized above, previous studies have primarily focused on PMA and revealed the PMA-dependent development of the RSN in preterm infants and the impact of gestational age (GA) at birth on its development.^11,32,33.45^ However, the effect of postnatal days has rarely been a focus. Clinically, the morbidity of neurodevelopmental impairments increases with a decrease in GA at birth. Cao et al.^33^ demonstrated that RSN in preterm infants mainly developed in the third trimester of gestation. The earlier preterm infants are born, the longer they need to stay in the hospital as a replacement for the mother’s womb. This may mean that the postnatal age (PNA) of very preterm infants spent outside the womb alters the development of synaptic organization, which results in atypical RSN. Consequently, postnatal day reflects a different time course for neuronal development than PMA. Although postnatal day is a key factor in neurodevelopment, as shown above, it has been difficult to evaluate the effect of postnatal day on infant development because the impact varies depending on the gestational weeks at birth, requiring many more neonates to be categorized. As such, one of the major limitations of previous studies is that they did not evaluate the impact of postnatal days on the development of RSN in the neonatal period. We presumed that the impact of postnatal day depending on GA can be evaluated by dividing preterm infants into groups based on GA for comparison with term neonates. This is feasible for neonates of various GAs, including very preterm neonates in our hospital. Consequently, the aim of this study was to evaluate the impact of postnatal days on the development of RSN in groups based on GA.

In this study, we examined RSNs in many infants with various GAs (22–41 weeks of GA) using functional near-infrared spectroscopy (fNIRS). Due to the limitations of channel numbers, we focused on connectivity in frontal–temporal–parietal regions. We examined differences in the effects of PMA and PNA on the RSNs of preterm infants with varying GA. The aim of this study was to explore the neural basis of the high morbidity of neurodevelopmental dysfunction in early preterm infants by examining the impact of GA, PMA, and PNA on cerebral connectivity.

## Methods

### Participants

The parents of the participants were approached for consent and enrollment between 2011 and 2018. This cross-sectional study included 32 term and 71 preterm neonates, each of whom contributed one data point without any longitudinal measurements. As shown in demographic data (Table 1), the participants were divided into three groups based on their GA at birth. In this study, preterm infants born at less than 30 weeks of gestation were classified as “low-GA preterm infants,” and preterm infants born at more than 30 weeks of gestation were classified as “high-GA preterm infants” This classification was based on previous findings, as summarized by Hand et al.,^47^ who reported that birth before 30 weeks of gestation is a significant predictor of brain impairment in preterm infants. An additional neonate was excluded because of insufficient data points (see criteria in Data Analysis) due to motion artifacts of rapid head movement and/or loose probe attachments. Among these three groups, almost all demographic characteristics such as GA and birth weight (Table 1) were significantly different (*P* < 0.001), with the exception of the PMA at the examination between low-GA preterm infants and term infants (*P* = 0.567). GA was determined by an obstetrician using data including the last menstrual period, the first accurate ultrasound examination, and assistive reproductive technology. We excluded infants with chromosomal or congenital anomalies, including congenital heart anomalies, grade 2–4 IVH, PVL, necrotizing enterocolitis, deafness diagnosed by automated auditory brainstem response, and those who were medically unstable. Ductus arteriosus was clinically closed at the time of examination in infants whose birth weight was >1500 g. In infants whose birth weight was <1500 g, closure of the ductus arteriosus was confirmed by echocardiography before the fNIRS measurement. This study was conducted at Keio University Hospital (Tokyo, Japan). The institutional review boards of the hospital approved all protocols related to the study, and informed consent was obtained from the parents of all participating infants.

**Table 1:** Characteristics of participating infants for the three groups.

	Preterm infants		Term infants
	Low-GA preterm	High-GA preterm	
	(*n* = 27)	(*n* = 44)	(*n* = 32)
GA, days, median (IQR)^†^	185 (168–197)	233 (223.75–240)	268 (262.75–279.25)
Birth weight, g, median (IQR)^†^	768 (632.5–1010.5)	1739.5 (1398.5–1984.25)	2994 (2557.5–3143.5)
Weight at measurement, g, median (IQR)^†^	2370 (2157–2649.5)	2035.5 (1891.5–2188.75)	2891 (2457–3148)
Postnatal age at the time of examination, days, median (IQR)^†^	88 (65.5–116)	20.5 (14.75–32)	4 (3–5)
Postmenstrual age at the time of examination, days, median (IQR)^†^	268 (257–288.5)	253.5 (250–259.5)	272.5 (267–282.5)
APGAR score at 1 min, median (IQR)^†^	3 (2–5)	7 (5.75–8)	9 (8–9)
APGAR score at 5 min, median (IQR)^†^	6 (6–7)	8 (8–9)	9 (9–9)

*GA* gestational age, *IQR* interquartile range.

^†^*P* < 0.001.

### Procedure

**Fig. 1:**
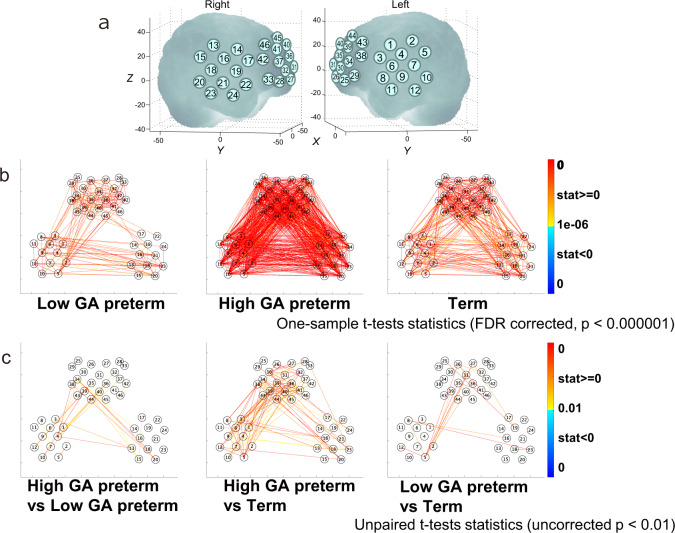
RSNs in different GA groups. Preterm infants born before 30 weeks of GA = “Low GA preterm”; preterm infants born after 30 weeks of GA = “High GA preterm”; term infants = “Term”. **a** Location of 46 channels on the infant’s head. Each channel is represented by a channel number. **b** RSN for each GA group. Significant RSNs are indicated by red and blue lines. One-sample *t* test statistics (FDR-corrected, *P* < 0.000001) for all networks. **c** Results of two-tailed unpaired *t* tests between each pair of groups (uncorrected). The red lines indicate stronger connectivity for high-GA preterm infants than for low-GA preterm infants (left panel), high-GA preterm infants than term infants (middle panel), and low-GA preterm infants than term infants (right panel).

fNIRS measurements were performed in a dim and quiet room at the hospital. Hemodynamic fluctuations were recorded at 46 positions in the bilateral temporal and frontal areas using fNIRS (ETG 4000, Hitachi Medical Corporation, Tokyo, Japan). Infants were tested when they were asleep. Because the experimenter paused the measurement whenever the infants showed a body movement that likely caused motion artifacts, the measurement period ranged from 4 to 7 min. A silicon pad with five incident and four detection probes, arranged in a 3 × 3 square lattice with a separation of 20 mm, was placed laterally on each side of the infant’s head (Fig. 1a). Each pad comprising 12 channels was attached to the head so that the center detector probe at the bottom of the horizontal probe line corresponded with the T3 or T4 position in the international 10/20 system, as described elsewhere.^48^ In addition, a pad of a 3 × 5 square lattice comprising eight incident and seven detection probes separated by 20 mm was attached to the frontal area. The vertical midline of the channels was positioned in alignment with the nasion-inion line, and the lowest horizontal probe line was set in a direction parallel to the T3-Fp1-Fp2-T4 line.

### Data analysis

Oxy- and deoxy-Hb concentrations were calculated using the modified Beer-Lambert law from the absorption of 695- and 830-nm laser beams sampled at 10 Hz. This transformation was performed using the platform for optical topography analysis tools (POTATo), which is a MATLAB (Math Works Inc., Natick, MA)-based analysis tool. In this procedure, the optical path length and absorption coefficients against oxy- and deoxy-Hb were assumed to be constant. The product of the optical path length (L) and the differential path length factor was set to 1 because the measured L was unavailable. The present study focused on the oxy-Hb signal, which is generally used for the analysis of RSN in fNIRS measurements for both adults and infants because of its higher signal-to-noise ratio.^49,50^ Furthermore, because most studies of infant RSN employed oxy-Hb or its equivalent signal with fMRI,^30–34,49,51,52^ the oxy-Hb index made it possible to easily compare with previous results on infant RSN. For artifact rejection, we followed the procedure of Imai et al.^51^ If there was a signal change of more than 0.15 mM·mm in total Hb (sum of oxy-Hb and deoxy-Hb) between the mean of four successive samples (i.e., 400 ms) and that of the next 4 successive samples, the last time point in the first four samples was marked as an artifact. The period between the 15 samples before and 85 samples after the time point (10 s) was marked as the artifact period. If the artifact period was simultaneously shared by more than 23 channels (50% of the total channels), all channels during that artifact period were marked as artifacts. After applying the third-order Butterworth bandpass filter (0.01–0.1 Hz), the time points of those marked artifacts were treated as missing values. The cut-off frequency of the high-pass filter (0.01 Hz) was determined based on the sample time points, which was approximately 210 s on average. Because 0.08 Hz is frequently used to eliminate cardiac and respiratory effects for the low-pass filter,^53^ we reanalyzed our data using another bandpass filter (0.01–0.08 Hz) to confirm our results. In principle, the results were consistent in terms of our study purposes (see Supplementary Materials for Supplementary Figs. 1 and 2).

For spatial estimation of the channel location in the brain, we employed a modified version of the virtual registration method to map fNIRS data onto the MNI standard brain space.^54^ This study used MRI template data from a single 12-month-old infant with macroanatomical segmentation and detailed landmarking of scalp structures.^55^ Specifically, this method linearly reduced the size of the infant template based on the head circumference (Fpz-T3-POz-T4-Fpz) of a 12-month-old infant and a neonate template.^52^ Based on the information of probe attachment in this study according to the international 10/20 system, macroanatomy of the lateral cortical surface was estimated primarily using the infant template with subsidiary reference to automatic anatomical labeling.^55,56^

### Functional connectivity

For each infant, Pearson’s correlation coefficients (*r*) on a pairwise basis were calculated between the time courses of oxy-Hb of all 46 measurement channels. Functional connectivity [*z* (*r*)] was obtained by applying Fisher’s *z*-transformation to the coefficients (*r*). For each channel pair within the group, individual *z* (*r*) values were averaged (i.e., functional connectivity) and examined using a one-sample *t* test against zero. The false discovery rate was applied to correct for multiple comparisons. We defined a criterion for valid connectivity with a sample size of more than 1200 time points (120 s). If the number of channel pairs that did not satisfy the criterion exceeded 776 (75% of the total 1035 pairs), the participant’s data were excluded. The average duration for all participants was 205.8 s (SD = 44.5, range: 120.0–315.0), and no significant difference among the groups was observed (*F* (2,100) = 1.67, *P* = 0.1942).

To examine the development of connectivity depending on the connectivity type including within-area and inter-area, we categorized the channels into three regions of interest: “frontal,” where the channels were in the prefrontal area, and “left” and “right,” where the channels were in the left and right temporal areas partly including the parietal area. In this procedure, anatomical information for each channel was based on the results of the virtual registration described above. Connectivity within each area (e.g., “frontal”) was defined as “within,” and connectivity across those areas (e.g., between “frontal” and “left”) was defined as “inter.” Averaged length of connectivity on the basis of the MNI template is 32.41 mm (SD15.68) and 71.26 mm (SD20.98) for “within” and “inter,” respectively, with significant difference between them (*t* = 31.52, *P* = 1e−152). Two-way ANOVAs were conducted on the connectivity, with GA (low-GA preterm, high-GA preterm, and term) as a between-subject factor and the area (“within” or “inter”) as a within-subject factor. We also performed regression analyses to examine the relationship between connectivity, PMA, and PNA for each group.

Finally, a statistical heteroscedastic comparison of the slopes of the two regression lines was performed on regression lines showing significance. This was performed using Student’s *t* test following the recommendations given by Andrade and Estévez-Pérez^57^ to compare the influence of PNA and PMA on connectivity between infants in the ≥30 GA group (high-GA preterm and term) and infants in the <30 GA group.

## Results

As shown in Fig. 1b, infants showed very strong connectivity for many channel pairs either between the frontal and temporal areas or between the right and left temporal areas. The three GA groups showed different patterns of RSN in these brain areas. In particular, high-GA preterm infants presented a relatively strong resting network relative to the other groups. The amplitude of connectivity was compared between each pair of groups, as shown in Fig. 1c. The results of the two-tailed unpaired t-tests between pairs of groups demonstrated that the RSN in high-GA preterm infants was stronger than that in low-GA preterm infants and term infants. In particular, the difference in the RSN appears to be marked in the inter-area networks. Low-GA preterm infants showed slightly stronger connectivity than term infants for some channel pairs, but they are not statistically significant.

**Fig. 2:**
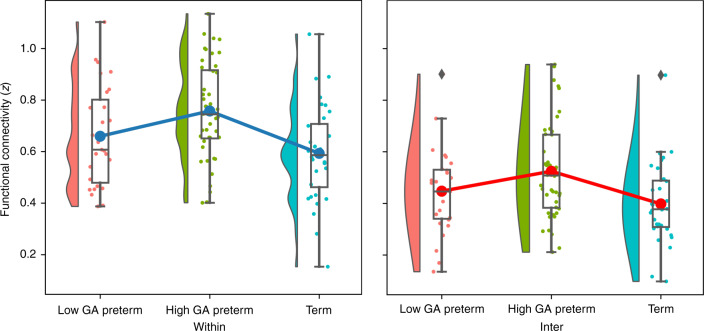
Box plots of amplitude for within- and inter-area connectivity (*z*-scores) and their frequency distributions in different GA groups. Preterm infants born before 30 weeks of GA = Low GA preterm; preterm infants born after 30 weeks of GA = High GA preterm; term infants = Term. Connectivity within frontal or temporal areas = Within; inter-area connectivity = Inter. Big dots in the center indicate averaged values.

Figure 2 illustrates the differences in RSN across groups and types of connectivity (within vs. inter). As shown in Fig. 2, within-area connectivity was stronger than inter-area connectivity. Connectivity in the high-GA preterm infants demonstrated the strongest connectivity among the groups. These tendencies were confirmed by two-way ANOVAs on the amplitude of RSN, with group as a between-subject factor and connectivity type (within and inter) as a within-subject factor. The results indicated significant main effects of group [*F* (2,100) = 6.67, *P* = 0.0019, *η*^2^ = 0.12] and connectivity type [*F* (1,100) = 460.93, *P* < 0.001, *η*^2^ = 0.82]. There was no significant interaction between group and connectivity type [*F* (2,100) = 1.4065, *P* = 0.24982, *η*^2^ = 0.27360]. Homogeneity of variance among the different groups was confirmed [*F* (2,100) = 0.38, *P* = 0.6871, *η*^2^ < 0.01) using Levene’s test. Because the group factor showed a significant effect on the RSN, we further analyzed the differences between each group using a post hoc test (Fisher’s least significant difference test). Significant differences were observed between the high-GA preterm infants and both the low-GA preterm infants (*t* (100) = 2.0558, *P* = 0.042, *d* = 0.41116) and term infants (*t* (100) = 3.58, *P* = 0.0005, *d* = 0.71).

Having demonstrated the significantly stronger connectivity in the high-GA preterm infants compared to that in the term infants, we presumed that the significant difference in both PNA and PMA at the examination between the two groups had affected the strength of the connectivity (*P* < 0.001 and *P* < 0.001, respectively; Table 1). To evaluate the correlation between connectivity and both PNA and PMA, a simple correlation analysis was performed by pooling the high-GA preterm group with either the term or low-GA preterm group. This was also used to assess whether groups showed a consistent tendency that would allow the optimal grouping of the two groups as one. This would also be effective in avoiding unreasonable analysis of the term group itself due to the limited range of PNA (3–5 days). For the high-GA and term groups, significant correlations between the amplitude of connectivity and PNA were observed in both within- and inter-area networks (*R* = 0.31, *P* = 0.005; *R* = 0.35, *P* = 0.0017, respectively) (Supplementary Fig. 3 left), while the simple correlation analysis between RSN and PMA did not demonstrate a significant difference in either within- or inter-area networks (*R* = −0.18, *P* = 0.10; *R* = −0.22, *P* = 0.05, respectively) (Supplementary Fig. 3 right). Regarding the pooling of high- and low-GA preterm infants, the results indicated no significant correlations for either PNA or PMA (*R* =−0.086, *P* = 0.47; *R* = −0.078, *P* = 0.52; *R* = 0.03, *P* = 0.75; *R* = −0.03, *P* = 0.75, respectively) (Supplementary Fig. 4).

**Fig. 3:**
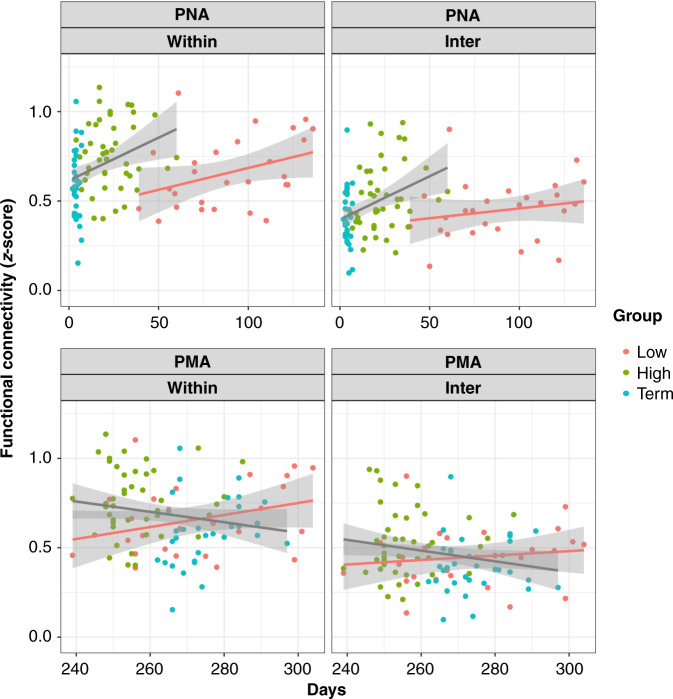
Comparison of the slopes of regression lines between the ≥30 GA and <30 GA groups. Two regression lines for the ≥30 GA and <30 GA groups are indicated for correlations of RSN (within and inter) and PNA (upper panel) and RSN and PMA (lower panel). Turquoise-blue circles indicate term infants. The moss-green circles indicate high-GA preterm infants. The pink circles indicate low-GA preterm infants. Two regression lines were drawn separately for infants born before 30 weeks of GA (<30 GA group) in pink and infants born after 30 weeks of GA (≥30 GA group) in gray.

**Fig. 4:**
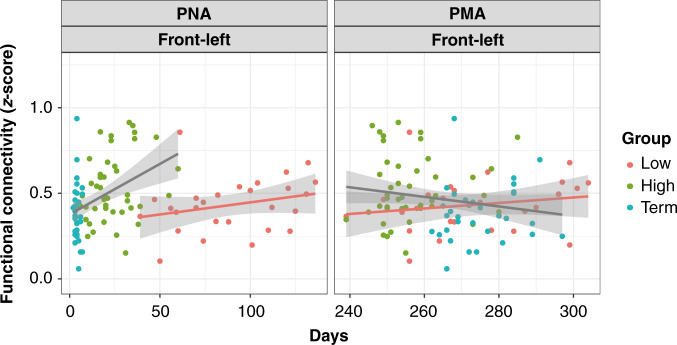
Comparison of the slopes of regression lines between the ≥30 GA and <30 GA groups. Two regression lines for the ≥30 GA and <30 GA groups are indicated for correlations of the frontal-left connectivity (network from the frontal area to the left temporal area) and PNA (left panel) and the frontal-left network and PMA (right panel). Connectivity from the frontal area to the left temporal area = Frontal-left.

Since we found a significant correlation between RSN and PNA upon pooling of the high-GA preterm infants and term infants, we hypothesized that these groups can be categorized into the same group. Consequently, we decided to reorganize infants into two groups based on GA (“≥30 GA group” and “<30 GA group”) by merging the term and high-GA preterm infants to examine the correlations between connectivity and PNA (Fig. 3). As shown in Fig. 3 (upper panel), the ≥30 GA group showed increased connectivity as a function of PNA, while the <30 GA group showed very gradual changes in connectivity. Statistical heteroscedastic comparison of the slopes of the two regression lines revealed a significantly steeper slope exclusively for the inter-area network in the ≥30 GA group than in the <30 GA group (*P* < 0.05) (Fig. 3). For further evaluation of specific connected brain areas, we performed statistical comparisons of the slopes of two regression lines in three different inter-area networks, namely, the frontal area to the left temporal area, the frontal area to the right temporal area, and the left to right temporal area. A significant difference in regression slopes and PNA was observed in a specific type of connectivity: the connectivity between the frontal and left temporal areas was different between the ≥30 and <30 groups (*P* < 0.05) (Fig. 4). No significant differences were observed between the other two networks.

## Discussion

One of the most urgent and important issues in current neonatal medicine with improved survival rates is to clarify the developmental mechanisms of preterm infants who are discharged from the hospital. This understanding will enhance our knowledge to guide such infants to optimal neurodevelopmental diagnosis or assistance. Accordingly, in this study, we examined the impact of PNA and PMA in preterm infants born at different gestation weeks on the development of their RSNs using fNIRS. By dividing the three infants into groups differing in GA, our results demonstrated that GA and PNA are both related to the development of the RSN. Although RSN generally develops as a function of PNA, as was the case in our data, its development differed depending on GA, as indicated by the significant difference in slope values. While infants born after 30 weeks of gestation, including high-GA infants and term infants, showed rapid development in inter-area connectivity according to PNA, low-GA preterm infants showed a very gradual increase as a function of PNA. Namely, the inter-area RSNs in the <30 GA group developed significantly slower than those in the group of infants whose GA was more than 30 weeks. Differences in their development were observed, especially in the networks across the frontal area to the left temporal area, including key networks involving language areas. These results indicate that the RSN of preterm infants born before 30 weeks of gestation was underdeveloped because of atypically slow development after birth. The present fNIRS study successfully captured developmental differences in RSNs in preterm infants depending on their GA. Optimal categorization of three groups enabled this analysis. Although our results are consistent with previous studies showing an underdeveloped RSN of very preterm infants, this study further revealed region-dependent development by pointing out slow development in connectivity between the frontal and left temporal areas exclusively in the <30 group.^7,32,35–40^ This may be one of the neural bases for the high morbidity of neurodevelopmental disturbances, including language delay, in very preterm infants.

The neurophysiological mechanisms underlying slow development in the inter-area RSN in low-GA preterm infants involve various factors that also differ depending on the individual clinical course before birth. However, this could possibly be explained by the altered development of immature neurovascular and/or metabolic systems in the cerebral cortex of very preterm infants. While it is well known that synaptic development occurs extensively during the 6 months after birth, capillary formation starts between term and 3 months of age. Well before the postnatal period at approximately 30 weeks of gestation, immature synaptic structures with less myelination and arteriole vessels and capillaries are gradually developing in the maternal uterus.^58–64^ More specifically, birth before 30 GA is a significant risk factor for brain impairments such as IVH and PVL.^47,65^ One of the neuronal bases of this has been said to be related to the subependymal geminal matrix, which is the source of neurogenesis and gliogenesis. By 30 weeks of gestation, neuronal migration ceases by switching to gliogenesis, resulting in the involution of the subependymal germinal matrix and development of the cortex.^66^ It is assumed that change into extrauterine environment during such unstable period without completion of neuronal migration may affect later brain development including brain connectivity. This means that the intrauterine environment may be suited for brain growth until the neuronal migration is complete, especially until the subependymal germinal matrix undergoes complete involution. It is to be noted that this tendency was observed for the low-GA preterm infants even without explicit brain injury. Recent studies examining the cerebral structure of preterm infants further revealed altered development in the neurovascular structure of very preterm infants compared to late preterm infants, which supports our results.^66,67^ Previous studies also demonstrated different RSNs between infants born in early gestation and infants born in later gestation at their expected due date, which is also consistent with our results.^7,32,35–40^ Our results further suggest that GA influences the postnatal development of a particular brain network. We speculate the existence of a critical period for neurogenesis in the maternal womb, which is optimal for the development of neural networks of fetuses, as exemplified by the involution of the germinal matrix previously mentioned. This is analogous to other premature complications. For instance, both the incidence and severity of retinopathy of prematurity, one of the major complications for very preterm infants, increases with decreasing GA.^68–72^ This is due to the abnormal vascularization of retinal vessels in very preterm infants in the extrauterine environment, similar to our study.

Consistent with our results, previous studies have demonstrated that very preterm infants at the expected date of birth presented weak or unorganized RSN compared to term infants.^7,32,35–40^ Although some researchers have investigated PMA-dependent development of RSNs in very preterm infants, it remains unclear why the RSN was still weak when the very preterm infants grew to their due date.^35–40,73^ In our study, we identified that the critical period for intrauterine growth that is optimal for the development of the RSN of fetuses is approximately 30 weeks of GA. If preterm infants are born before 30 weeks of GA, they have underdeveloped networks even at their due date because of the slow PNA-dependent development of RSNs. On the other hand, in the case of birth after approximately 30 weeks of gestation, PNA seems to have a positive impact on the RSNs, as observed from stronger connectivity in high-GA preterm infants. It is assumed that once the basic cortical structure is organized to a particular level after the completion of neurogenesis, an extrauterine environment with various sensory and social stimuli (e.g., speech, face, and haptic input) can better enhance neurodevelopment, particularly for inter-area networks. This interpretation is supported by accumulated evidence on how environmental factors influence preterm neonates and term neonates.^74–76^ Previous neuroimaging studies on neonates rarely examined factors of PNA due to the limited number of participants and restricted variations of PNA and GA. Among such limited studies, neurovascular development in neonates was reported to be dependent on PNA but progressed at a slower rate in early preterm infants, which is consistent with our results.^77^ Such PNA-dependent development of hemodynamic or metabolic systems could also contribute to the formation of neuronal networks, as observed in our study. Future studies should further examine angiogenesis and synaptogenesis depending on PNA and PMA, because other studies have reported that mature neurovascular coupling of a hemodynamic response is associated with PMA^78^ and weight at examination.^79^ We also examined the effect of weight (see Supplementary Fig. 5) on RSN. The results were similar to those obtained for PMA, and there was no clear correlation for the ≥30 GA group.

Another crucial factor related to the development of RSNs in preterm infants is the connectivity type and brain region. While the present study demonstrated significantly slower development of inter-area RSNs in the <30 GA group than in the ≥30 GA group, no significant differences were observed for the within-area RSN. While the development of region-dependent RSN in preterm infants has been shown to be different from that of term infants,^30–35,73^ many reported weaker and slower development of inter-area or inter-hemispheric connectivity in preterm infants.^30,32,80,81^ Regardless of GA, younger infants, including neonates, tend to show strong and dense RSN for short-range and/or within-area connectivity, and inter-area connectivity gradually develops until adulthood.^49,79,82^ Our results are therefore consistent with these previous studies in that young infants showed weaker connections for inter-area than for within-area, and PNA strengthened inter-area RSN in general. However, our results further extended previous findings by categorizing three groups to take GA, PNA, and PMA into account and revealed a relationship between GA and PNA-dependent development of inter-area RSN. Such inter-area or long-range connections are neuronal bases of cognitive function,^82^ which are constructed and strengthened through cognitive experiences in the development of cognition. Therefore, slower development of inter-area RSN in the <30 GA group may explain the higher prevalence of future neurodevelopmental disturbances, including language function, in very preterm infants.^2–4,83–85^ Indeed, atypical development of the left frontal-temporal connection in very preterm infants found in our study as well as in Kwon et al.^30^ may affect later language development because that connection covers the language network.

The interpretation of the results of this study is limited by the variability in the clinical information of the participants. Clinical indices such as cardiovascular and respiratory status might affect the long-term outcomes of preterm infants.^86–89^ Although the present study showed significant differences in the effect of PNA on the development of RSN in preterm infants based on their GA, not all of the clinical information in the participants was considered for analysis. However, our study excluded participants with major complications (see “Methods” for details). Our exclusion criteria for participants were similar to those of previous studies and were stricter than those of other studies.^11,32,33,45^ Although it is assumed that the clinical course of very preterm infants differs from that of late preterm infants, our results demonstrated that even in the absence of major complications, the impact of PNA on the development of the RSN in very preterm infants is different from that in late preterm infants.

The limitations of this study include the restricted brain regions that were measured and the analyzed parameters (oxy-Hb within 0.01–0.1 Hz). The fNIRS methodology does not allow whole-brain measurements, which prevented us from analyzing RSNs for each functional brain region.^8,46^ Nevertheless, our results clearly demonstrated that the development of RSN in preterm infants varies depending on the region, even within limited areas. As for the analysis of oxy-Hb within a particular frequency range, a recent study reported the robustness of total-Hb measures.^90^ Since infants’ hemodynamic physiology remains unclear in terms of the relationship between oxy- and deoxy-Hb and young or premature infants tend to show reversed Hemodynamic Response Function (HRF)^78,79^ and differences in hemoglobin phase,^91^ we do not have much evidence of what total-Hb measure reflects for functional brain networks. Consequently, in this study, our discussion is limited to the results of the conservative analysis. However, analysis of RSN with fNIRS has a unique potential for its accessibility to three hemodynamic measures, and the use of frequency-dependent analysis in addition to additional Hb measures (e.g., total-Hb) would reveal new evidence. By maximizing fNIRS, future studies should employ variable parameters to uncover neural development, including angiogenesis and synaptogenesis, which support functional brain networks.

## Supplementary information

Supplementary information
