# Supplementary material for: Differential age-dependent development of inter-area brain connectivity in term and preterm neonates
Source: Pediatr Res. 2022 Jan 29;92(4):1017–25. doi: 10.1038/s41390-022-01939-7 (PMC9586860; doi:10.1038/s41390-022-01939-7)
Supplement: Supplementary file 1 — Supplementary information [file 41390_2022_1939_MOESM1_ESM.docx]

Supplementary Figures


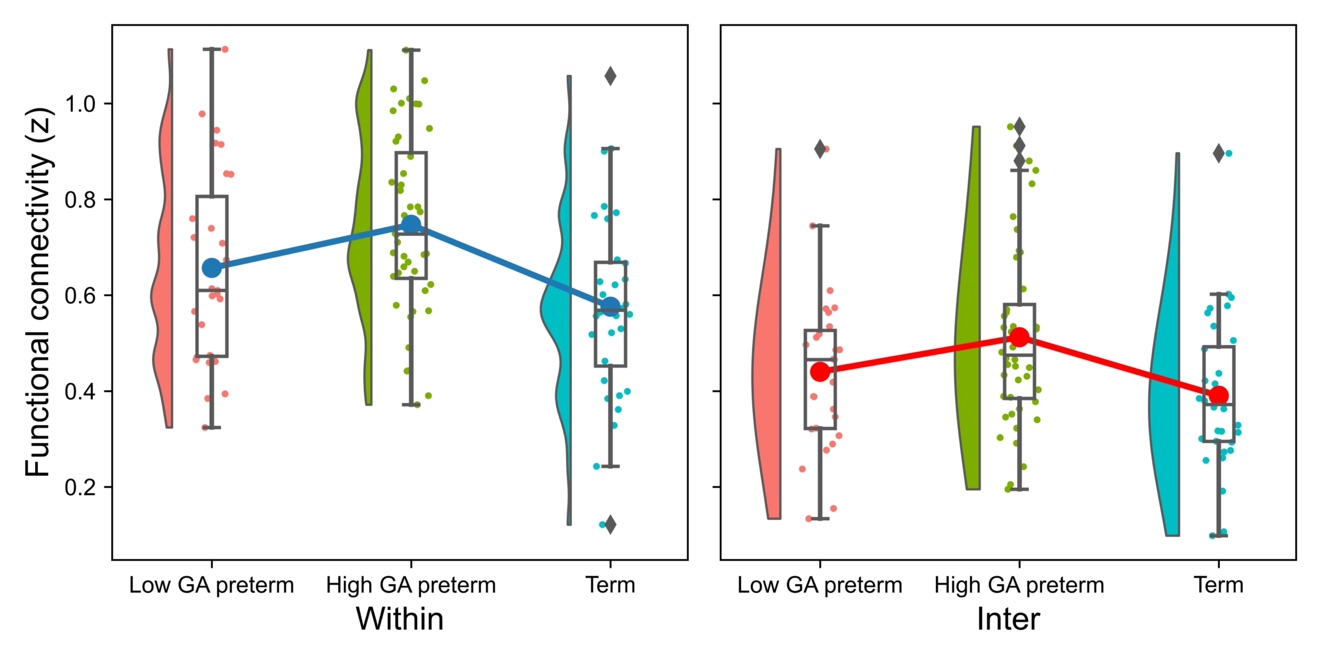


Suppl. Fig1. Box plots of amplitude for within- and inter-area connectivity (z-scores) in different GA groups (data preprocessed with 0.01-0.08Hz band pass filter)

Preterm infants born before 30 weeks of GA = “Low GA preterm”; preterm infants born after 30 weeks of GA = “High GA preterm”; term infants = “Term”. Connectivity within frontal or temporal areas = “Within”; connectivity inter-areas = “Inter”.


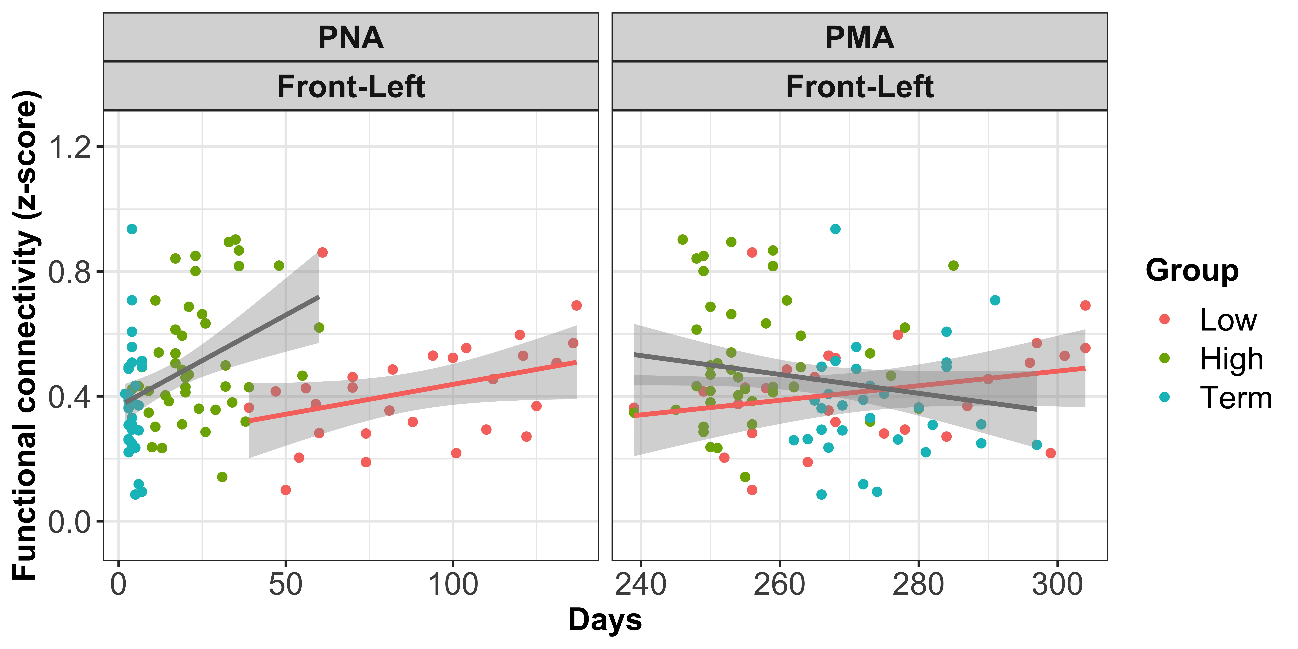


Suppl.Fig2.

Comparison of the regression analysis between the ≥30 GA and <30 GA groups (data preprocessed with 0.01-0.08Hz band pass filter)

Two regression lines for the ≥30 GA and <30 GA groups are indicated for correlations of the frontal-left connectivity and PNA (left) and the frontal-left network and PMA (right). Connectivity from the frontal area to the left temporal area = “Frontal-left”.


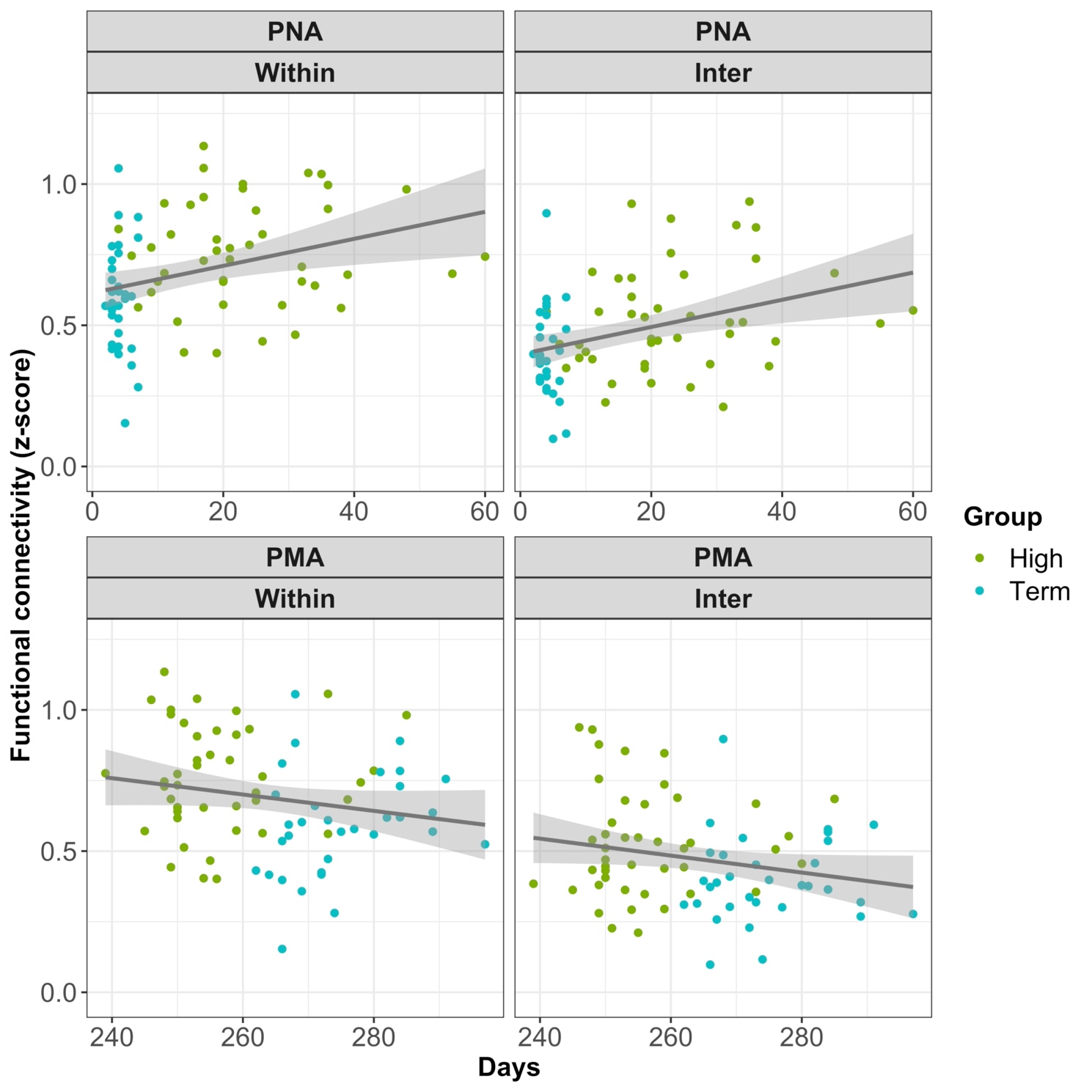


Suppl.Fig.3.

Simple regression analyses between RSN and, PNA and PMA.

Correlation between amplitude of connectivity and both PNA (upper) and PMA (lower) in high-GA preterm infants and term infants. Moss-green circle indicates high-GA preterm infants. Turquoise-blue circle indicates term infants. Connectivity within frontal or temporal area =“Within”, connectivity inter-areas =“Inter”.


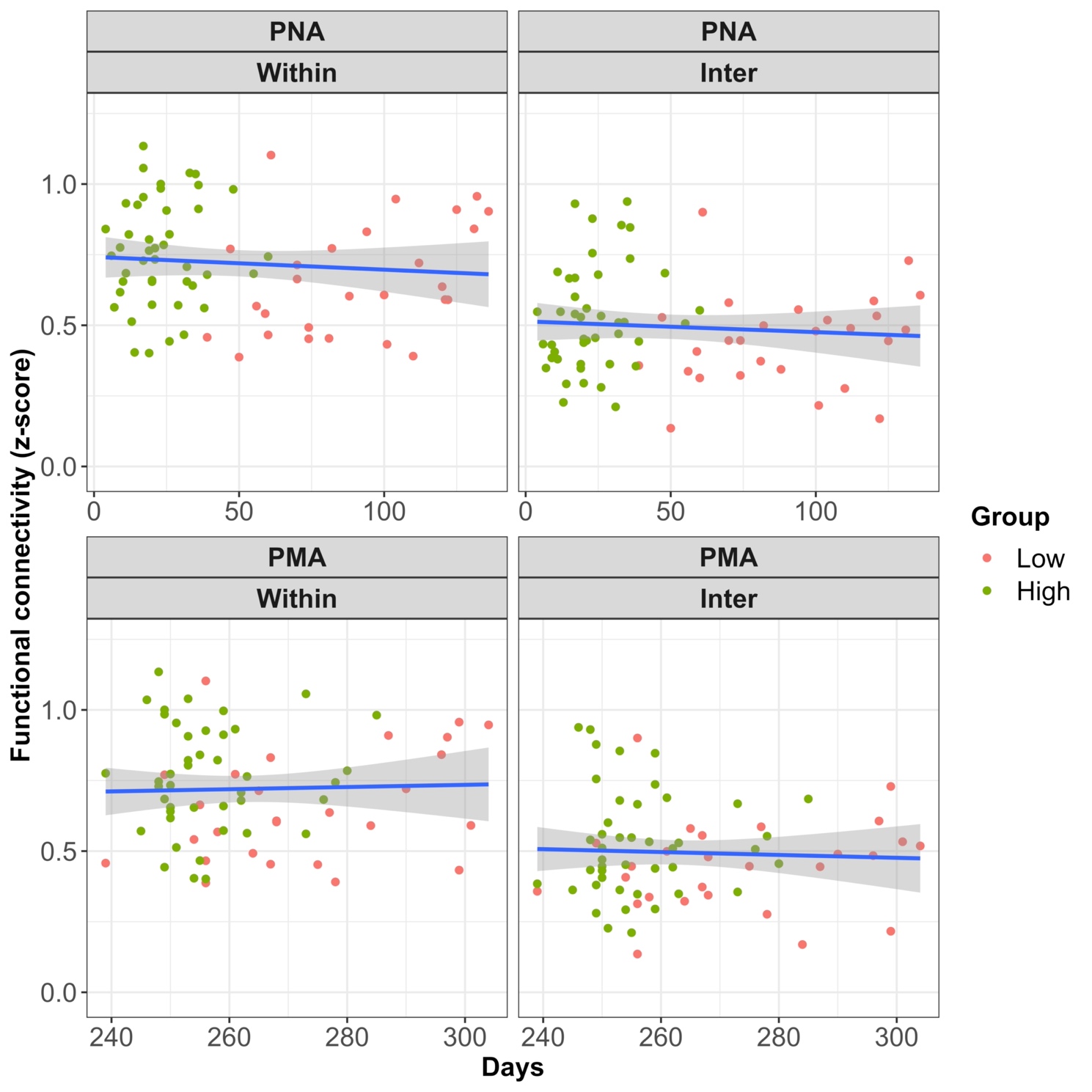


Suppl. Fig. 4. Simple regression analyses between RSN and, PNA and PMA

Correlations between amplitudes of connectivity and both PNA (upper) and PMA (lower) in high-GA preterm and low-GA preterm infants. Moss-green circles indicate high-GA preterm infants. Pink circles indicate low-GA preterm infants. Connectivity within frontal or temporal areas = “Within”; connectivity inter-area = “Inter”.


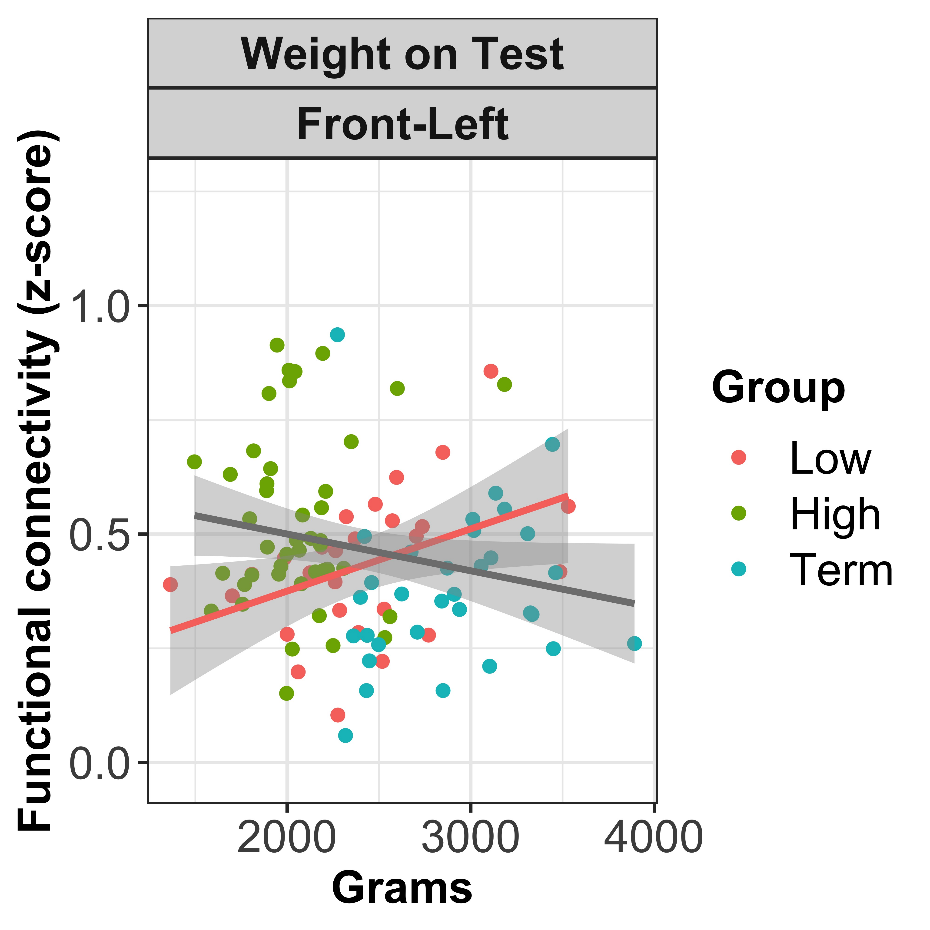


Supple. Fig5

Comparison of the regression analysis between the ≥30 GA and <30 GA groups

Two regression lines for the ≥30 GA and <30 GA groups are indicated for correlations of the frontal-left connectivity and weight at examination.
